# Multivariate normal tissue complication probability modeling of gastrointestinal toxicity after external beam radiotherapy for localized prostate cancer

**DOI:** 10.1186/1748-717X-8-221

**Published:** 2013-09-23

**Authors:** Laura Cella, Vittoria D’Avino, Raffaele Liuzzi, Manuel Conson, Francesca Doria, Adriana Faiella, Filomena Loffredo, Marco Salvatore, Roberto Pacelli

**Affiliations:** 1 Institute of Biostructures and Bioimaging, National Council of Research (CNR), Naples, Italy; 2 Department of Advanced Biomedical Sciences, Federico II University School of Medicine, Naples, Italy; 3 Department of Physics, University Federico II, Naples, Italy

## Abstract

**Background:**

The risk of radio-induced gastrointestinal (GI) complications is affected by several factors other than the dose to the rectum such as patient characteristics, hormonal or antihypertensive therapy, and acute rectal toxicity. Purpose of this work is to study clinical and dosimetric parameters impacting on late GI toxicity after prostate external beam radiotherapy (RT) and to establish multivariate normal tissue complication probability (NTCP) model for radiation-induced GI complications.

**Methods:**

A total of 57 men who had undergone definitive RT for prostate cancer were evaluated for GI events classified using the RTOG/EORTC scoring system. Their median age was 73 years (range 53–85). The patients were assessed for GI toxicity before, during, and periodically after RT completion. Several clinical variables along with rectum dose-volume parameters (Vx) were collected and their correlation to GI toxicity was analyzed by Spearman’s rank correlation coefficient (Rs). Multivariate logistic regression method using resampling techniques was applied to select model order and parameters for NTCP modeling. Model performance was evaluated through the area under the receiver operating characteristic curve (AUC).

**Results:**

At a median follow-up of 30 months, 37% (21/57) patients developed G1-2 acute GI events while 33% (19/57) were diagnosed with G1-2 late GI events. An NTCP model for late mild/moderate GI toxicity based on three variables including V65 (OR = 1.03), antihypertensive and/or anticoagulant (AH/AC) drugs (OR = 0.24), and acute GI toxicity (OR = 4.3) was selected as the most predictive model (Rs = 0.47, p < 0.001; AUC = 0.79). This three-variable model outperforms the logistic model based on V65 only (Rs = 0.28, p < 0.001; AUC = 0.69).

**Conclusions:**

We propose a logistic NTCP model for late GI toxicity considering not only rectal irradiation dose but also clinical patient-specific factors. Accordingly, the risk of G1-2 late GI increases as V65 increases, it is higher for patients experiencing previous acute toxicity and it is lower for patients who take AH/AC drugs. The developed NTCP model could represent a potentially useful tool to be used in prospective trial and for comparison among different RT techniques.

## Background

Prostate cancer (PC) is the most frequent non-cutaneous malignancy diagnosed in males in Europe. For localized disease 3D-conformal radiotherapy (RT) coupled or not with hormone therapy represents a very effective treatment. Most patients experience long lasting disease free survival and eventually are cured from PC. However, long term gastrointestinal (GI) toxicity is a feared drawback of RT treatment. The commonly reported GI complications include rectal bleeding, fecal incontinence and changes in bowel habits. Owing to its objectivity, rectal bleeding was one of the most frequently, or even exclusively, reported rectal toxicity [1]. However, the Quantec report [1] encouraged to separately score and model different and specific aspects of rectal toxicity. This becomes more significant in view of the decreasing toxicity profile due to the evolution of radiation therapy treatment technology in PC radiotherapy [2-5]. In addition, even nonbleeding morbidity such as mild or moderate urgency, frequency, diarrhea-like stools and fecal incontinence may significantly affect patient quality of life. Accordingly, parameters for Lyman-Kutcher-Burman (LKB) model of normal tissue complication probability (NTCP) for a number of different GI toxicity endpoints and scale have been recently reported [6].

The risk of GI complications has been shown to be affected by several factors other than the radiation dose to the rectum such as baseline patient risk factors (advanced age, smoking habits, diabetes, previous abdominal surgery), hormonal or antihypertensive therapy, and acute rectal toxicity [7-10]. To establish tailored strategies for a patient-adapted RT, models that take into account relationships among different patient-related and dosimetric factors may offer a powerful approach to the optimization of risk ascertainment for many different endpoints [7,9,11-15]. As a consequence, data-driven multivariate modeling of normal tissue complication probability (NTCP) [16] may offer a powerful and clinically useful approach to the GI toxicity risk evaluation unlike traditional NTCP models that only involve dose distribution parameters of a specific organ at risk like the LKB model.

The aim of the present study is to investigate the impact of the several clinical factors proposed in the literature along with dosimetric variables on the risk of developing GI late toxicity in patients receiving radiation therapy for PC. Multivariate logistic NTCP modeling exercise was performed using bootstrapping together with performance comparison with a traditional dose-based model for mild/moderate GI toxicity.

## Methods

### Patients

Data on 57 consecutive patients with localized prostate adenocarcinoma treated with radiation therapy at the Radiation Oncology department of the University "Federico II" of Naples were retrospectively reviewed. The patient’s median age was 73 years (range 53–85). Median prostate specific antigen (PSA) at diagnosis was 12.5 ng/ml (range 2.3–228). Twenty patients were classified as low risk (T1-2, PSA < 15, Gleason ≤ 6). All clinical information as cardiac comorbidities, diabetes, previous abdominal surgery, smoking history, hormonal therapy, and drugs prescription were retrieved from medical records.

### Radiotherapy and dosimetric analysis

All patients were treated with full three-dimensional radiation treatment planning delivered with 20 MV photon beams from linear accelerator by conformal radiation technique (CRT) or by conformal dynamic arc radiation technique (ART). CRT was administered using six coplanar beam arrangement at gantry angles of 45°, 90°, 135°, 225°, 270°, and 315°. Wedges were used for the oblique beams. All fields were shaped to the projection of the planning target volume (PTV) in the beam’s-eye view for conformal therapy. XIO (Elekta CMS) treatment planning systems was used. ART was delivered using two-dynamic lateral conformal arcs (40°–140° and 220°–320°) with dynamic modification of the leaves position. The ERGO (3D-Line Medical System) treatment planning system and micromultileaf collimator (leaves of 5 mm at isocenter) were used. For both CRT and ART, dose distribution was calculated using an algorithm appropriate for heterogeneous tissues.

Treatment planning was based on computed tomography (CT) performed with empty rectum, comfortably filled bladder, and with the patient in prone position using vacuum-locked mattress. Five-millimeter increment CT slices of the pelvis extending from L4-L5 to 2 cm caudal to the bottom of ischial tuberosities were acquired. CT images were electronically transferred to the CT simulation software (Focal Ease 4.2, Elekta CMS) for target and critical organs contouring. Clinical target volume (CTV) included the prostate gland or the prostate gland plus the seminal vesicles. A 1-cm margin was 3D automatically added around the CTV to define the PTV, except at the boundary between the anterior rectal wall and the prostate where a 0.5-cm margin was used. The rectum delineation was performed on purpose by the same radiation oncologist (M.C.) according to the male Radiation Therapy Oncology Group (RTOG) Normal Pelvis Atlas [17]. The prescription dose was specified at the center of the PTV. Field weightings were adjusted to achieve 95% of prescription dose to 95% of the PTV. A total dose of 66 Gy to seminal vesicles and 76 Gy to the prostate gland with daily fractions of 2 Gy (5 times per week) was planned.

Rectum dosimetric parameters were extracted from the dose-volume histograms (DVH) for modeling. Dosimetric parameters included: the maximum (Dmax) and mean doses (Dmean), the percentage volume exceeding 20–75 Gy (Vx) in increment of 5 Gy.

### Recording of GI toxicity

Acute GI treatment toxicity (toxicity present during radiotherapy and in the first 3 months thereafter), and late GI toxicity (follow-up >3 months) was evaluated by physicians according to Radiation Therapy Oncology Group/European Organization for Research and Treatment of Cancer (RTOG/EORTC) criteria [18] as summarized in Table 1. The patients have been monitored for GI toxicity as part of the clinical routine before, during, and periodically after RT completion. Follow-up visits were planned every 3 months for the first year, then every 6 months for the next 3 years, and yearly thereafter. Patients who experienced one of the RTOG/EORTC endpoint prior to RT treatment (i.e. irritable bowel disorders) were excluded from the analysis.

**Table 1:** **Acute and late GI morbidity scoring system according to RTOG/EORTC scale**[18]

**Acute morbidity**				
Grade	I	II	III	IV
	Increased frequency or change in quality of bowel habits not requiring medication/rectal discomfort not requiring analgesics	Diarrhea requiring drugs/mucous discharge not necessitating sanitary pads/rectal or abdominal pain requiring analgesics	Diarrhea requiring parenteral support/severe mucous or blood discharge necessitating sanitary pads/abdominal distention	Acute or subacute obstruction, fistula or perforation; GI bleeding requiring transfusion; abdominal pain or tenesmus
**Late morbidity**				
Grade	I	II	III	IV
	Mild diarrhea; mild cramping	Moderate diarrhea and colic	Obstruction or bleeding requiring surgery	Necrosis/Perforation
	Bowel movements 5 times daily	Bowel movements >5 times daily		Fistula
	Slight rectal discharge or bleeding	Excessive rectal mucus or intermittent bleeding		

### Statistical modeling

Dosimetric parameters along with patient clinical covariates reported in the existing literature were included in the analysis. Univariate logistic analysis for each parameter was performed using the Spearman’s rank correlation (Rs) coefficient to assess correlation with late GI risk.

In order to identify combinations of variables that were likely to be most predictive of GI toxicity, we used automated logistic regression with bootstrap technique for variable selection, and bootstrap resampling to test selection stability. We used 500 bootstraps for each analysis. The logistic regression model is defined as

(1) $$\mathrm{NTCP}=\frac{g\left(x\right)}{1+e^{g\left(x\right)}}$$

with

(2) $$g\left(x\right)=\beta_{0}+\beta_{1}x_{1}+\beta_{2}x_{2}+\ldots \beta_{n}x_{n}$$

Where *x*_*1*_*, x*_*2*_*.…. x*_*n*_ represent different input variables and *β*_*0*_*, β*_*1*_*.…. β*_*n*_ are the corresponding regression coefficients.

When the correlation coefficient between two variables was greater than 0.75, in order to avoid overfitting, we removed the one with the lowest correlation with GI toxicity from the subsequent multivariate analysis.

Data analysis was performed by an open source available package (Dose Response Explorer System, DREES [16]) as described in detail in previous publications [13,19]. Model predictive power is quantified using Rs correlation coefficient while the area under the curve (AUC) of receiver operating characteristic (ROC) curve was used to evaluate the discriminating ability of model fits. The discrimination value on the ROC curve was determined by Youden’s J statistic. Statistical analysis was performed using MedCalc (MedCalc, Mariakerke, Belgium).

For comparison purpose, we also calculated the NTCP values using the LKB dose-based model for specific rectal complications proposed by Gulliford et al. [6]. We used the parameters reported for Grade 1 and Grade 2 nonbleeding endpoints. Different models were compared using the AUC values.

## Results

Twenty-one out of 57 patients (36.8%) experienced an event of acute GI toxicity of grade G1 or G2 after the end of the radiation treatment. At a median follow-up of 30 months (range 6–112) after the end of the radiation treatment and with 90% of patients being free of biochemical relapse, 19 out of 57 patients (33.3%) developed late GI toxicity of grade G1 or G2. It should be noted that the more common symptoms were high stool frequency, loose stools and rectal urgency while no rectal bleeding was recorded for these patients.

Table 2 summarizes the available clinical variables, patient and treatment characteristic as well as the summary of dosimetric statistics for the rectum. The results of the univariate logistic regression analysis for clinical and dosimetric parameters were also reported in Table 2.

**Table 2:** Clinical variables, summary of dosimetric statistics and correlation coefficient (Rs) with radiation-induced late gastro-intestinal toxicity incidence

			**Univariate analysis**	
**Characteristic**			**Rs**	**p-value**
*Categorical*	*N*	*%*		
Age (y)				
≤70	19	33.3		
>70	38	66.7	0.105	.436
Tumor size				
<T3	48	84.2		
≥T3	9	15.8	-0.044	.752
PSA				
≤15	35	61.4		
>15	22	38.6	0.051	.707
Gleason score				
≤6	34	59.7		
>6	23	40.3	0.143	.315
Hormonal therapy				
Yes	53	7.0		
No	4	93.0	-0.097	.472
Previous abdominal surgery				
Yes	26	45.6		
No	31	54.4	0.025	.854
Diabetes				
Yes	14	24.6		
No	43	75.4	-0.058	.670
Smokers				
Yes	31	54.4		
No	26	45.6	0.199	.137
Antihypertensive/anticoagulants				
Yes	37	64.9		
No	20	35.1	-0.338	.010
Acute GI toxicity				
Yes	21	36.8		
No	36	63.2	0.309	.020
Radiation treatment				
CRT	26	45.6		
ART	31	54.4	-0.174	.195
*Continuous*	*Median (range)*			
Dmax (Gy)	76.2 (47.2-79.7)		-0.009	.947
Dmean (Gy)	43.2 (14.9-74.9)		0.242	.035
V30 (%)	67.1 (16.8-100)		0.203	.131
V40 (%)	49.4 (12.3-100)		0.244	.067
V50 (%)	39.8 (8.2-99.1)		0.274	.039
V60 (%)	30.1 (2.6-98.0)		0.282	.034
V65 (%)	27.0 (0.4-98.2)		0.284	.032
V70 (%)	26.8 (0.0-97.0)		0.255	.056
V75 (%)	7.6 (0.0-81.7)		0.068	.615

**Figure 1:**
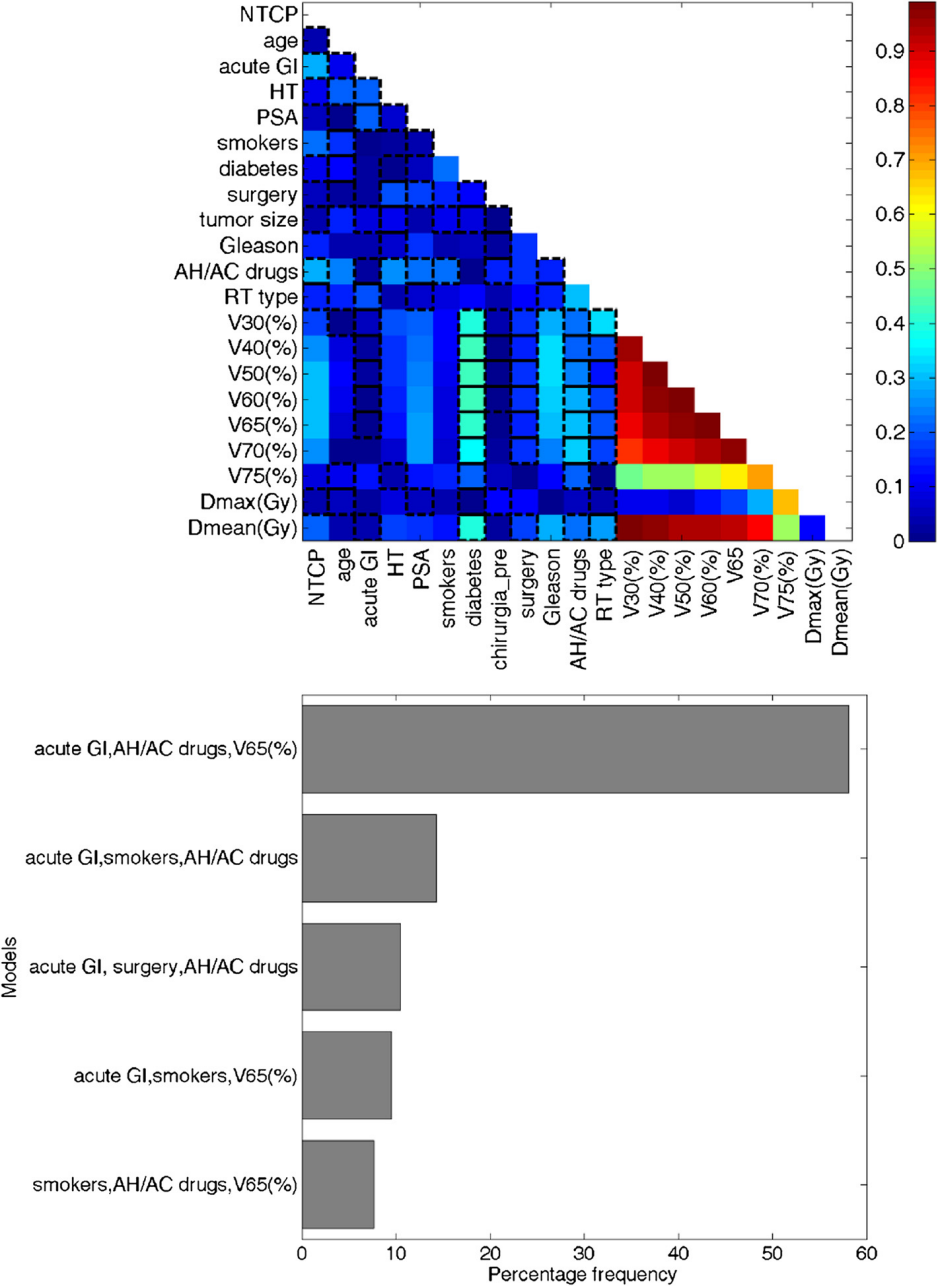
**Cross-correlation matrix (a) and the five most frequently selected models by bootstrap sampling technique (b).** The lateral bar represents the Spearman’s rank correlation coefficient value. NTCP: normal tissue complication probability; HT: hormonal therapy; PSA: prostate specific antigen; AC/AH: antihypertensive/anticoagulants; RT: radiation therapy; Vx (%): percentage of rectum volume exceeding X Gy.

Figure 1a shows the cross-correlation matrix for clinical and dosimetric variables. A strong multiple correlation between dosimetric parameters was found. Accordingly, these highly correlated variables were not included in the multivariate analysis.

**Figure 2:**
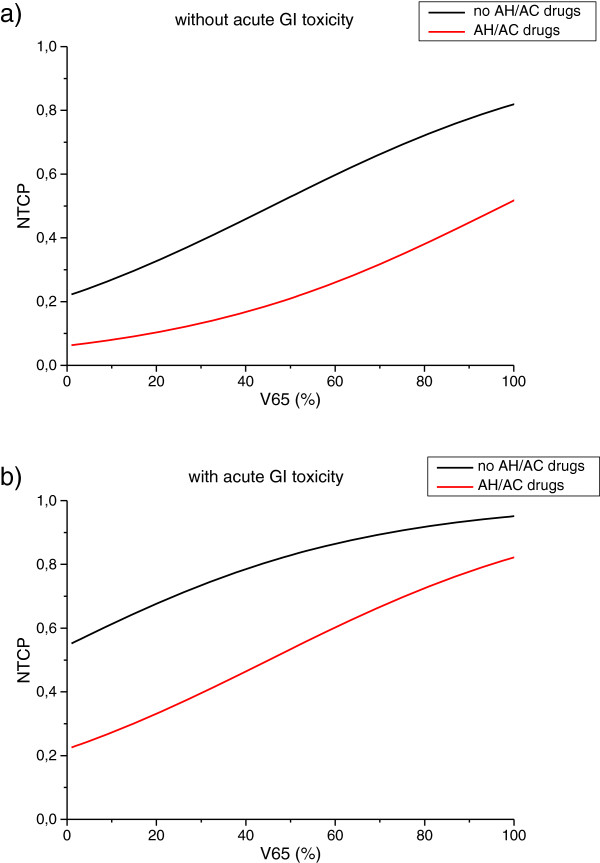
Three-variable NTCP model curves as a function of V65 for patients who experienced gastro-intestinal (GI) acute toxicity (a) and for patients who did not (b).

A three-variable model was suggested as the optimal order by bootstrap method. Figure 1b shows the five most frequently selected models within the bootstrapped subpopulations. The optimal model (*model 1*) includes V65, antihypertensive and/or anticoagulant (AH/AC) drugs use (yes = 1, no = 0) and previous acute toxicity (yes = 1, no = 0). The Spearman’s rank correlation coefficient of the model is 0.47 (p < 0.001) and the AUC of the corresponding ROC curve is 0.79. The best-fitted regression coefficients are given in Table 3. According to this model, the risk of late GI toxicity of grade G1 or G2 increases as V65 increases, it is higher for patients experiencing previous acute toxicity and it is lower for patients who take AH/AC drugs. *Model 1* NTCP curves are represented in Figure 2a-b. In Table 3 the regression coefficients for the logistic model based on V65 only (*model 2*) are also reported. The Rs coefficient of *model 2* is 0.28 (p < 0.001). The result of ROC analyses was a discrimination value for V65 of 29.3%.

**Table 3:** Best-fitted regression coefficients for NTCP models and odds ratios (OR)

	**Parameter**	**Estimated coefficient**	**SE**	**p-value**	**OR**
*Model 1*					
	V65 (%)	0.028	0.017	0.052	1.03
	Antihypertensive/anticoagulants	-1.442	0.669	0.031	0.24
	Acute GI toxicity	1.458	0.669	0.029	4.30
	constant	-1.283			
*Model 2*					
	V65 (%)	0.033	0.016	0.036	1.03
	constant	-1.702			

**Figure 3:**
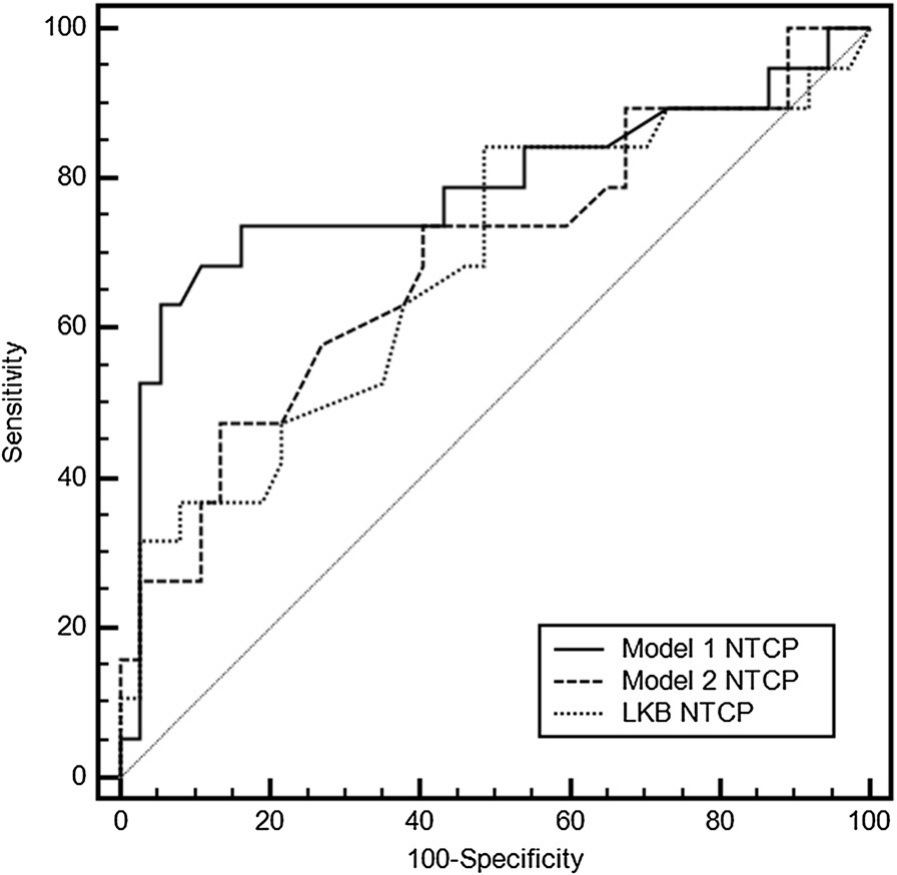
Comparison of receiver operator characteristic (ROC) curves obtained applying three-variable NTCP model (model 1), V65-based NTCP model (model 2) and LKB NTCP model.

**Figure 4:**
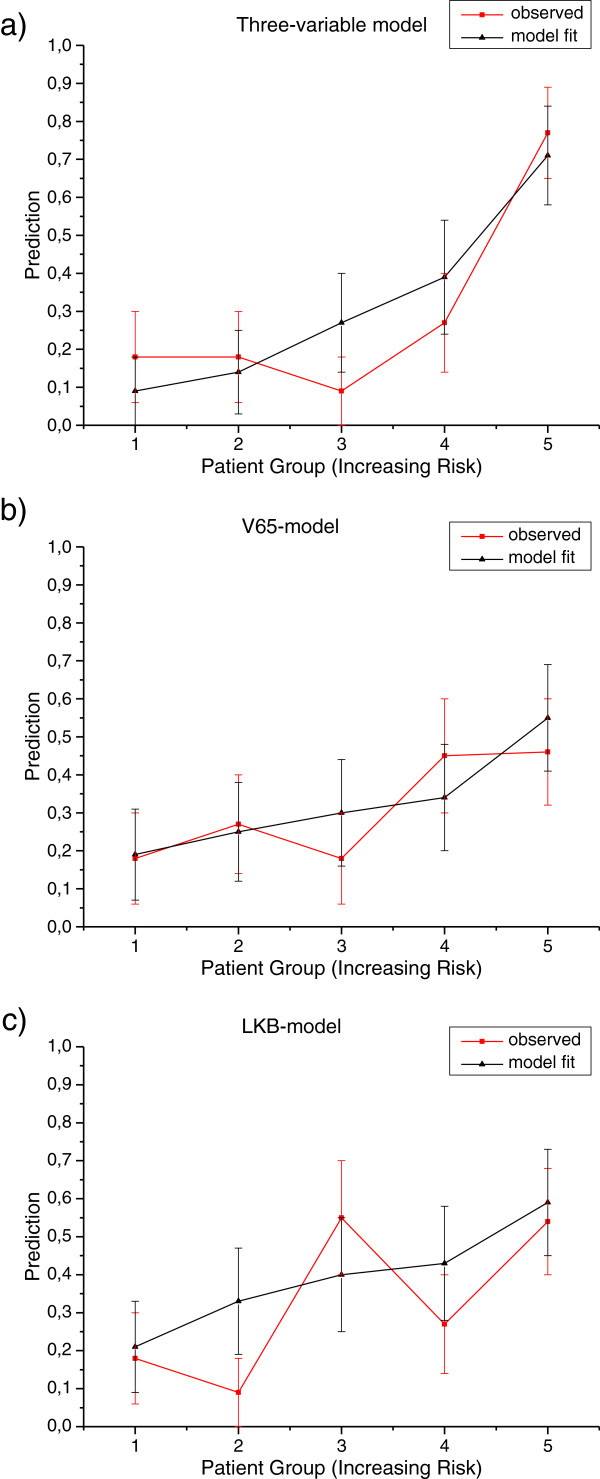
**Mean predicted rates of GI toxicity vs. observed rates for patients binned by predicted risk.** The patients are binned, with equal patients number in each bin, according to the three-variable NTCP model **(a)**, to the V65-based NTCP model **(b)**, and to the LKB NTCP model **(c).** Continuous line: observed risk; dot lines: prediction model.

In Figure 3 the comparison is reported among the ROC curves obtained applying *model 1*, *model 2* and *LKB model*. The AUC values were 0.79, 0.69 and 0.68, respectively. The comparison of the predicted incidence of GI toxicity by each of the above models and the actuarial incidence in the population is shown in Figure 4.

## Discussion

External beam 3D conformal radiotherapy represents an effective option to cure localized prostate cancer with most patients experiencing long lasting freedom of relapse survival. On the other hand, the anatomic proximity of the rectum to the prostate gland causes an exposition to high doses of radiation for this part of the GI tract with consequent risk of late toxicity able to significantly affect the patients quality of life. Thus, in the process of treatment planning, dose constraints for given rectal volumes are recommended as a conservative starting point for 3D treatment planning [1]. However, the dose and its fractionation [20,21] are not the only actors in the determinism of toxicity risk. Several patient associated features have been shown to be potential predictive factors impacting on late GI toxicity in prostate cancer RT [8-10,15,22]. The inclusion of these factors in the toxicity prediction model could be very helpful to obtain a more personalized plan evaluation a given patient. In addition, different types of symptoms in the rectum after prostate cancer RT have been identified, namely rectal bleeding, fecal incontinence, urgency and frequency. Each of these symptoms is likely to be caused by diverse pathogenic mechanisms [23]. Accordingly, different predisposing clinical variables may have a different impact on GI toxicity. Clinically useful NTCP models should be developed separately for each endpoint [6,7].

In the present study we have explored the feasibility of building an effective multivariate logistic NTCP model for late RTOG gastrointestinal toxicity prediction in PC patients undergoing radiation therapy. We used bootstrap validation to determine the combination of variables that generated the highest true predictive performance. When only limited samples are available, the bootstrap method involves generating a number of resamples of an observed dataset. The size of each of these resamples is equal to the observed dataset and is obtained by random sampling with replacement from the original dataset [14,24]. The advantage of the applied procedure is that the use of Spearman’s rank correlation coefficient allows a very effective identification of the relatively stronger combination of the variables able to predict a definite outcome.

In our unselected population of prostate cancer patients, at a median follow-up of 30 months, we found an incidence of GI late toxicity of grade ≤ 2 of 33.3%. This result is substantially comparable to the incidence of late rectal toxicity reported by Skala et al. [4] on a cohort of 443 patients. They found 27.7% of G1-G2 late GI toxicity and 0.7% of G3 toxicity. Of note, in our population we did not record any rectal bleeding. As consequence, the endpoints for which the NTCP was modeled are alterations of intestinal motility and peristalsis such as high stool frequency, loose stools and rectal urgency.

Through multivariate logistic regression we obtained a 3-variable NTCP model with a good predictive power (AUC = 0.79). This model, in addition to the dose-volume parameter V65, includes patient specific variables such as acute GI toxicity after RT and use of antihypertensive and/or anticoagulants drugs.

The obtained three-variable logistic NTCP model (*model 1*) was compared using the AUC of the ROC curves with the logistic model based only on V65 (*model 2*) and with the LKB NTCP model (Figure 3). Based on the AUC analysis, no difference in performance was found between *model 2* and the *LKB model*. Conversely, *model 1* outperforms the logistic model based on conventional DVH data only or the traditional LKB dose-based model. The improved performance of *model 1* can be also observed from the larger risk ratios between the highest-risk bins and the lowest risk bins when classified by model predictions (Figure 4a). The high ratio indicates that this model might be a useful clinical model.

For mild/moderate radiation-induced nonbleeding rectal toxicity we found a dose-volume constraint of 29.3% for V65 that is comparable with the 25% reported from the Quantec reviews [1] for moderate/severe toxicity.

The benefits of including clinical factors in rectal toxicity prediction after RT for prostate cancer has already been demonstrated [7-10] and in particular Valdagni et al. [9] proposed and validated a set of nomograms for prediction of late rectal toxicity using as endpoints G2-G3 rectal bleeding and fecal incontinence. However, the type of toxicity for which our multivariable NTCP model is proposed is milder compared with that described in the above study. In our cohort, the late GI toxicity mostly consisted in high stool frequency and loose stool that, even if milder than fecal incontinence and rectal bleeding, have a considerable impact on quality of life of prostate cancer survivors.

Our results are in agreement with the study by Fellin et al. [8] who reported the impact of clinical variables on moderate/severe persistent fecal incontinence. In particular, the use of antihypertensive drugs has been found to act like a protective factor while the acute severe incontinence was the most predictive parameter. Heemsbergen et al. [25] also found that acute GI toxicity was an independent significant predictor of late GI toxicity.

The statistical evidence that antihypertensive drugs act as protective factor does not surprise considering the clinical study by Kharofa et al. [26] that strongly suggests that the use of angiotensin-converting enzyme (ACE) inhibitors is a protective factor against radiation induced pneumonitis in patients undergoing thoracic irradiation. Furthermore, a preclinical study [27] shows the protective activity of ACE inhibitors on irradiated brain tissue in rats.

The endpoints modeled in our study are possible consequences of microvascular radiation damage in the submucosa related to colitis cystica profunda [23], and at the same time the antihypertensive drugs have been suggested to have a vascular protective effect and the ability to regress the vascular remodeling [28].

Correlations between acute and late effects of irradiation have been reported in a number of tissues, mainly in the urinary and intestinal systems [29]. This phenomenon, known as consequential late effect, is defined as a direct consequence of acute radiation response causing tissue damage and probably leading to late effects after a latent symptom-free interval.

With the caveats of the low number of analyzed patients and the relatively short follow-up time, the strength of our study is that patients were all homogeneously treated, with one physician contouring the rectum, and evaluated in a single Institution. Of note, the rate of toxicity was comparable with other published prostate cancer series [4,6]. Beyond the main difference of using mild toxicity endpoints rather than the most common moderate/severe complications, our methods and findings have been already touched by many research groups [8,24,30,31]. However, bootstrap and leave-one-out methods in NTCP modeling, parameters for non-bleeding late GI toxicity, AH/AC drugs as effective protecting drugs, and the inclusion of clinical factors other than dose have been often dealt with separately in the literature. The present modeling exercise have covered all the above mentioned aspects in order to put the results into a clinically useful perspective. The developed NTCP model could represent a potentially useful tool to be used in prospective trial for comprehensive comparison among different emerging RT techniques in the treatment of prostate cancer, and to explore the expected effects from rectum dose-volume reduction [5,32]. Indeed, as Figure 2 shows, the same dose reduction to the rectum could imply a different estimation of toxicity risk in different patients being individual clinical factors, such as AH/AC therapy, crucial for the prediction of toxicity risk.

## Conclusions

In conclusion, in the present study we propose a logistic NTCP model for predicting mild to moderate late rectal toxicity through a data-driven multivariate modeling approach and through the inclusion of comprehensive patients related factors plus rectal dosimetric parameters. Our results demonstrate that the combination of antihypertensive and/or anticoagulant drugs and previous acute toxicity improve prediction capability of NTCP models for late radio-induced GI toxicity. The proposed model represents an additional tool towards a tailored radiation therapy plan evaluation.

## Abbreviations

AH/AC: Antihypertensive and/or anticoagulant; ART: Arc radiation technique; AUC: Area under the curve; CRT: Conformal radiation technique; CTV: Clinical target volume; EORTC: European organization for research and treatment of cancer; GI: Gastrointestinal; LKB: Lyman-Kutcher-Burman; NTCP: Normal tissue complication probability; PC: Prostate cancer; PSA: Prostate specific antigen; PTV: Planning target volume; ROC: Receiver operator characteristic; Rs: Spearman’s rank correlation; RT: Radiation therapy; RTOG: Radiation therapy oncology group.

## Competing interests

The authors declare that they have no competing interests.

## Authors’ contributions

LC, and RP conceived and designed the study. AF, FD, FL, MC, MS reviewed patient clinical and dosimetric data. LC, RL, VDA performed statistical modeling and analysed the data. All authors participated in drafting and revising the manuscript. All authors have given their final approval of the manuscript.
